# Kinetics of Neutralizing Antibody Response Underscores Clinical COVID-19 Progression

**DOI:** 10.1155/2021/9822706

**Published:** 2021-10-19

**Authors:** Qing Lei, Hongyan Hou, Caizheng Yu, Yandi Zhang, Jo-Lewis Banga Ndzouboukou, Xiaosong Lin, Zongjie Yao, Hui Fu, Ziyong Sun, Feng Wang, Xionglin Fan

**Affiliations:** ^1^Department of Pathogen Biology, School of Basic Medicine, Tongji Medical College, Huazhong University of Science and Technology, Wuhan, China; ^2^Department of Laboratory Medicine, Tongji Hospital, Tongji Medical College, Huazhong University of Science and Technology, Wuhan, China; ^3^Department of Public Health, Tongji Hospital, Tongji Medical College, Huazhong University of Science and Technology, Wuhan, China

## Abstract

**Background:**

Neutralizing antibody (nAb) response is generated following infection or immunization and plays an important role in the protection against a broad of viral infections. The role of nAb during clinical progression of coronavirus disease 2019 (COVID-19) remains little known.

**Methods:**

123 COVID-19 patients during hospitalization in Tongji Hospital were involved in this retrospective study. The patients were grouped based on the severity and outcome. The nAb responses of 194 serum samples were collected from these patients within an investigation period of 60 days after the onset of symptoms and detected by a pseudotyped virus neutralization assay. The detail data about onset time, disease severity and laboratory biomarkers, treatment, and clinical outcome of these participants were obtained from electronic medical records. The relationship of longitudinal nAb changes with each clinical data was further assessed.

**Results:**

The nAb response in COVID-19 patients evidently experienced three consecutive stages, namely, rising, stationary, and declining periods. Patients with different severity and outcome showed differential dynamics of the nAb response over the course of disease. During the stationary phase (from 20 to 40 days after symptoms onset), all patients evolved nAb responses. In particular, high levels of nAb were elicited in severe and critical patients and older patients (≥60 years old). More importantly, critical but deceased COVID-19 patients showed high levels of several proinflammation cytokines, such as IL-2R, IL-8, and IL-6, and anti-inflammatory cytokine IL-10 in vivo, which resulted in lymphopenia, multiple organ failure, and the rapidly decreased nAb response.

**Conclusion:**

Our results indicate that nAb plays a crucial role in preventing the progression and deterioration of COVID-19, which has important implications for improving clinical management and developing effective interventions.

## 1. Introduction

In December 2019, an emerging severe acute respiratory syndrome coronavirus 2 (SARS-CoV-2) was first identified as the pathogen of coronavirus disease 2019 (COVID-19) [1]. As of 19 March 2021, there have been more than 121 million confirmed COVID-19 patients and 2.68 million deaths worldwide [2]. Up to the present, the COVID-19 pandemic has evolved as the most serious threat to global public health, social, and economic development. The spectrum of SARS-CoV-2 infections is broad. Asymptomatic [3] and symptomatic pneumonia with different severities rang from mild and moderate to severe and critical conditions that require intensive care and invasive ventilation [4–7]. Clearly, it is of great significance to understand how host immunity underlines the progression of SARS-CoV-2 infections for improving clinical management, formulating effective interventions, and designing efficacious vaccines.

Neutralizing antibody (nAb) has been generally thought to play an important role in the protection against a broad of viral infections, such as SARS-CoV [8], Ebola virus [9], and H5N1 avian influenza virus [10]. A large amount of growing evidence showed that nAb might also prevent SARS-CoV-2 infection. For example, several highly potent nAbs targeting the spike (S) protein on the viral envelope of SARS-CoV-2 have been isolated from patients and explored the efficacy for prevention or treatment of COVID-19 in various animal models or clinical trials [11–16]. A study conducted in nonhuman primates showed that nAb could confer protection against reexposure of SARS-CoV-2 [17]. nAb also has been routinely detected after vaccination with COVID-19 vaccine candidates in preclinical and clinical trials [18–22]. However, the results of immunotherapy with convalescent plasma collected from severe COVID-19 cases remained controversial [23, 24], although severe COVID-19 patients tended to have higher levels of nAb than mild patients [25–29]. Only low levels of nAb were detected in asymptomatic SARS-CoV-2 infections and vanished in a short time [30]. Reinfection has raised concerns that immunity from previous infections may be transient [31, 32]. In addition, several studies reported that nAb responses may be more easily induced in older [29, 33–35] or male [33, 35, 36] patients. nAb responses in COVID-19 patients peaked in the days following the onset of symptoms and declined over time [27, 29, 33, 37, 38]. Despite these remarkable advances, the role of nAb underlining the clinical progression of COVID-19 remains poorly understood.

To fully understand the kinetics of nAb response and their clinical significance during clinical COVID-19 progression, 123 COVID-19 cases in this retrospective study were randomly selected from 1056 hospitalized patients in Tongji Hospital during the epidemic. Their serum samples were collected, and the levels of nAb in each serum were detected by a pseudotyped virus neutralization assay. nAb responses underlining the disease progression were further established by correlating the levels of nAb with the onset time, severity of illness, strategies of clinical treatment, laboratory biomarkers, and clinical outcomes, respectively. Our research indicates that nAb plays a crucial role in preventing the progression and deterioration of COVID-19 patients.

## 2. Materials and Methods

### 2.1. Patient Information and Clinical Data Sources

123 COVID-19 patients with different severities of illness and outcomes were randomly selected from 1056 patients in Tongji Hospital, Wuhan, China, between 17 February 2020 and 28 April 2020 [30]. Patients with COVID-19 were diagnosed based on positive RT-qPCR results for detecting SARS-CoV-2 nucleic acid from respiratory tract specimens or based on clinical diagnosis with clinical symptoms and imaging features of pneumonia on chest computed tomographic (CT) according to the fifth version of COVID-19 diagnostic and treatment guideline published by the National Health Commission of China (NHCC). According to the guideline, COVID-19 patients with different severity of illness were classified into four groups. (1) Mild cases have mild clinical symptoms and no sign of pneumonia on imaging. (2) Moderate cases have clear clinical symptoms such as fever and other respiratory symptoms and chest imaging of pneumonia. (3) Adult severe cases meet any of the following criteria: (a) respiratory distress (≥30 breaths/min), (b) oxygen saturation ≤ 93% at rest, (c) arterial partial pressure of oxygen (PaO2)/fraction of inspired oxygen (FiO2) ≤ 300 mmHg (l mmHg = 0.133 kPa), and (d) chest imaging that shows obvious lesion progression (>50%) within 24-48 hours. (4) Critical ill cases match any of the following criteria: (a) respiratory failure and requiring mechanical ventilation, (b) shock, and (c) other organ failure that requires ICU care.

The detail information of each case about demographic information, medical history, signs and clinical symptoms, chest CT, laboratory findings, treatments with clinical progression, and clinical outcome was collected from electronic medical records. Laboratory biomarkers related with the disease severity such as lymphocytes, D-dimer, and C-reactive protein (CRP) were performed by automated analyzers according to the manufacturers' instructions. The level of IL-6 in serum was measured by the electrochemiluminescence method (Roche Diagnostics).

### 2.2. Ethical Approval

The study was approved by the Ethical Committee of Tongji Hospital, Tongji Medical College, Huazhong University of Science and Technology, Wuhan, China (IRB ID: TJ-C20200128).

### 2.3. Serum Specimens

Serum specimens were collected from each of 123 patients during hospitalization, and a total of 194 samples were obtained and stored at -80°C until use. The serum specimens were inactivated at 56°C for 30 minutes before the detection, and all operations were carried out in a BSL-2 microbiology laboratory.

### 2.4. Pseudotyped Virus Neutralization Assay

Serum nAb detection was conducted based on a pseudotyped virus neutralization assay system as described previously [30]. In brief, the SARS-CoV-2 pseudovirus neutralization assay was carried out on Vero E6 cells (CRL-1586) in a 96-well plate. 50 *μ*L of serial 2-fold diluted sera from 1 : 10 to 1 : 2560 from each serum sample was prepared, and equal volumes of SARS-CoV-2 pseudovirus were added, and the plates were preincubated at 37°C for 1 h. 24 h before infection, 100 *μ*L of 10^4^ Vero E6 cells was added into each well of a 96-well plate. After washed and added 100 *μ*L fresh culture medium, cells were incubated with 100 *μ*L of sera-pseudovirus mixture for 48 h. The cells were collected with 200 *μ*L of digestion solution and used to determine the number of eGFP-expressing cells by FACS. The positive rate of eGFP-expressing cells (PRG) was calculated after collected 1000 cells, and the results were shown as PRG_pesudovirus with serum_. Vero E6 cells treated with the pseudovirus alone or with culture medium alone were used as positive or negative controls. The positive rates for controls were expressed as PRG_pesudovirus_ and PRG_blank_, respectively. Experiments were repeated twice. The neutralization rate (%) for different dilutions was calculated as the following:

(1) $$\begin{array}{c} \text{Neutralization rate }\left(\%\right)=\frac{\left({\text{PRG}}_{\text{pesudovirus}}-{\text{PRG}}_{\text{pesudovirus with serum}}\right)}{\left({\text{PRG}}_{\text{pesudovirus}}-{\text{PRG}}_{\text{blank}}\right)}\times 100\%. \end{array}$$

The titer of nAb was expressed as the half-maximal neutralizing titer (NT50), which was determined as the highest dilution ratio of each serum with 50% neutralization rate and calculated by using nonlinear regression in SPSS. The results were shown as the medians of NT50 and interquartile ranges (IQRs) of different groups.

### 2.5. Statistical Analysis

All statistical analyses were performed using Prism 8, SPSS, and R softwares. Loess regression model was used to determine the kinetics of nAb. Differences among different groups were conducted by two-way ANOVA, Mann-Whitney *U* test, Wilcoxon matched-pairs signed rank, Kruskal-Wallis test, and post hoc test (Dunn-Bonferroni), when required. Statistical significance was determined when a value of *p* < 0.05.

## 3. Results

### 3.1. Kinetics of Neutralizing Antibody Response in COVID-19 Patients

123 COVID-19 patients (60 females and 63 males) were enrolled in the study. Baseline characteristics of all participants were obtained from electronic medical records and shown in Table S1. Based on disease severity and outcome, the patients were divided into four groups, namely, mild/moderate (*n* = 68), severe (*n* = 26), critical-recovered (*n* = 11), and critical-deceased cases (*n* = 18), respectively.

**Figure 1:**
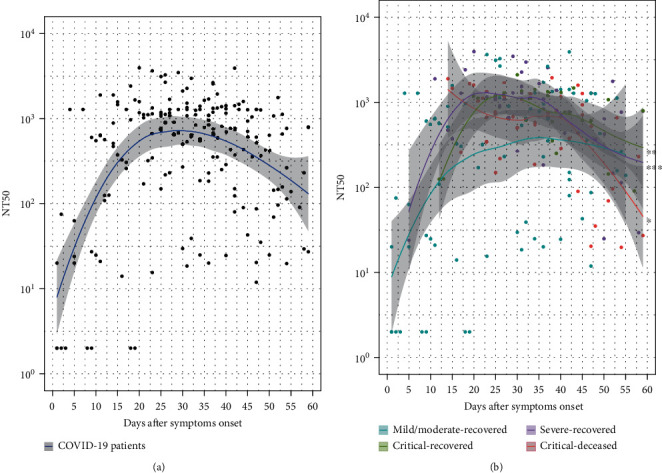
Kinetics of neutralizing antibody response in COVID-19 patients. Based on the disease severity and outcome from electronic records, 123 patients were classified into four groups, namely, 68 mild/moderate, 26 severe, 11 critical-recovered, and 18 critical-deceased cases. 194 serum samples were collected from these patients. Neutralizing antibody was detected for each sample based on the pseudotyped virus neutralization assay. The results were expressed as the half-maximal neutralizing titer (NT50). The line shows the mean value expected from a Loess regression model, and the ribbon indicates the 95% confidence interval. To visualize the data, a value of NT50 below 1 : 10 for the sample was plotted at a NT50 = 2. (a) The dynamic change of neutralization antibody response in COVID-19 patients after the onset of symptoms (*n* = 194). (b) Dynamic changes of neutralization antibody response among COVID-19 patients with different severity and outcome.

A total of 194 serum samples were collected from these patients. The dynamic changes of serum nAb response for each patient during the investigation period of 60 days after the onset of symptoms are shown in Figure 1. Consistent with previous reports [29, 33, 38], nAb response in symptomatic COVID-19 patients apparently experienced three stages, namely, rising, stationary, and declining phases (Figure 1(a)). More specifically, nAb response evolved as early as the first day following the onset of symptoms and then rose progressively. 20 to 40 days later, the response peaked and retained stably with the NT50 value approaching 1 : 1000. After 40 days, the level of nAb began to decrease. Until the end of experiments, the value of NT50 was about 1 : 200.

Interestingly, differential dynamic changes of the nAb response were further demonstrated at different stages among the patients of four groups (Figure 1(b)). During the rising period, the mild/moderate patients had the slowest increasing rate of nAb of four groups. In the stationary phase, both severe and critical patients tended to induce strong nAb response compared to mild/moderate patients. During the declining stage, critical-deceased patients decreased the fastest of all groups.

### 3.2. Influencing Factors of Neutralizing Antibody Response in COVID-19 Patients

**Figure 2:**
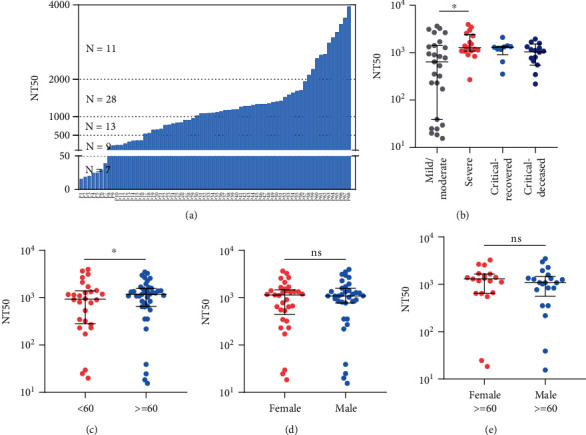
The effects of disease severity, age, and gender on the neutralizing antibody response. Serum samples during the stationary phase (20 to 40 days after symptoms onset) were collected from 68 patients and used for the detection of neutralizing antibody. The results were expressed as the NT50. Median and interquartile range value for each group were indicated. Differences between groups were analyzed using the Mann-Whitney *U* test. Differences among four groups were analyzed using Kruskal-Wallis test and post hoc test (Dunn-Bonferroni). (a) NT50 values of 68 COVID-19 patients. (b) Comparison of NT50 values among four groups of patients. Mild/moderate: *n* = 27, severe: *n* = 17, critical-recovered: *n* = 9, and critical-deceased patients: *n* = 15. (c) Comparison of NT50 values between age < 60 (*n* = 28) and age ≥ 60 (*n* = 40). (d) Comparison of NT50 values between female (*n* = 33) and male (*n* = 35). (e) Comparison of NT50 values between female with age ≥ 60 and male with age ≥ 60. ^∗^*p* < 0.05; ns means no statistical difference.

To further analyze the influencing factors of the nAb response, we chose serum samples of the stationary phase (20 to 40 days after symptoms onset) from 123 enrolled patients for further nAb assessment. These serum samples were obtained from a total of 68 cases (Table S2). Interestingly, all of these patients generated positive nAb responses. In particular, 76.5% (52/68) patients evolved strong nAb responses and the NT50 value was more than 1 : 500. 11.5% (7/68) patients induced low levels of nAb, and their NT50 values were less than 1 : 50 (Figure 2(a)). More importantly, severe (NT50 = 1 : 1280), critical-recovered (NT50 = 1 : 1302), and critical-deceased patients (NT50 = 1 : 1079) trended to elicit strong nAb responses, when compared to mild/moderate patients (NT50 = 1 : 640) (Figure 2(b)). Consistent with other reports [29, 33–35], older patients (≥60 years old) elicited higher levels of nAb than the younger (Figure 2(c)). Different from other reports [33–35], there was no statistical difference of the nAb response between male and female groups (Figure 2(d)) and between males and females over the age of 60 (Figure 2(e)), respectively. Therefore, our results indicate that age and disease severity of the patients affect the magnitude of serum nAb responses.

### 3.3. Longitudinal Changes of Neutralizing Antibody Response in COVID-19 Patients

**Figure 3:**
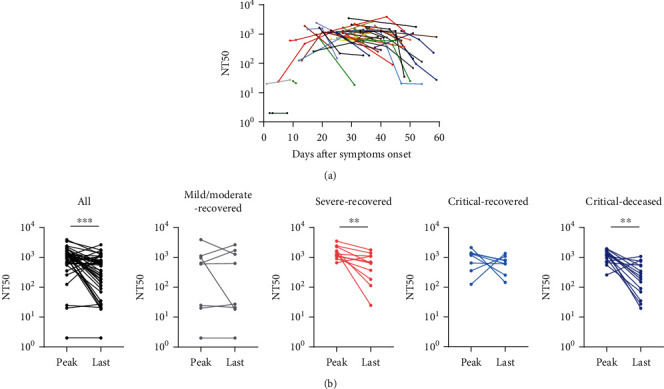
Longitudinal changes of neutralizing antibody response in COVID-19 patients. 42 COVID-19 patients with serial serum samples (more than one sample) were chosen for the detection of neutralizing antibody. The results were expressed as the NT50. (a) Longitudinal changes of neutralizing antibody response for each patient. Different colors of the lines mean the responses of each patients. (b) Comparison of the peak value of NT50 during hospitalization with that of the last serum from each patient before discharge or death. Mild/moderate: *n* = 8, severe: *n* = 11, critical-recovered: *n* = 8, and critical-deceased patients: *n* = 15. Differences between groups were analyzed using Wilcoxon matched-pairs signed rank. ^∗∗^*p* < 0.01; ^∗∗∗^*p* < 0.001.

Based on available serial serum samples, 42 cases (Table S3) were further chosen from the total of 123 patients to demonstrate longitudinal changes of the nAb response. As shown in Figure 3(a), the nAb response also appeared to have three stages over the time after symptoms onset, in agreement with our previous results. The geometric mean value of NT50 of the last serum from each patient before discharge or death decreased sharply, compared with the peak value of NT50 during hospitalization in these patients (Figure 3(b)). In particular, the nAb response of 69% patients (29/42) decreased over time, irrespective of disease severity and outcome.

### 3.4. Longitudinal Changes of Neutralizing Antibody Response with Clinical Progression

**Figure 4:**
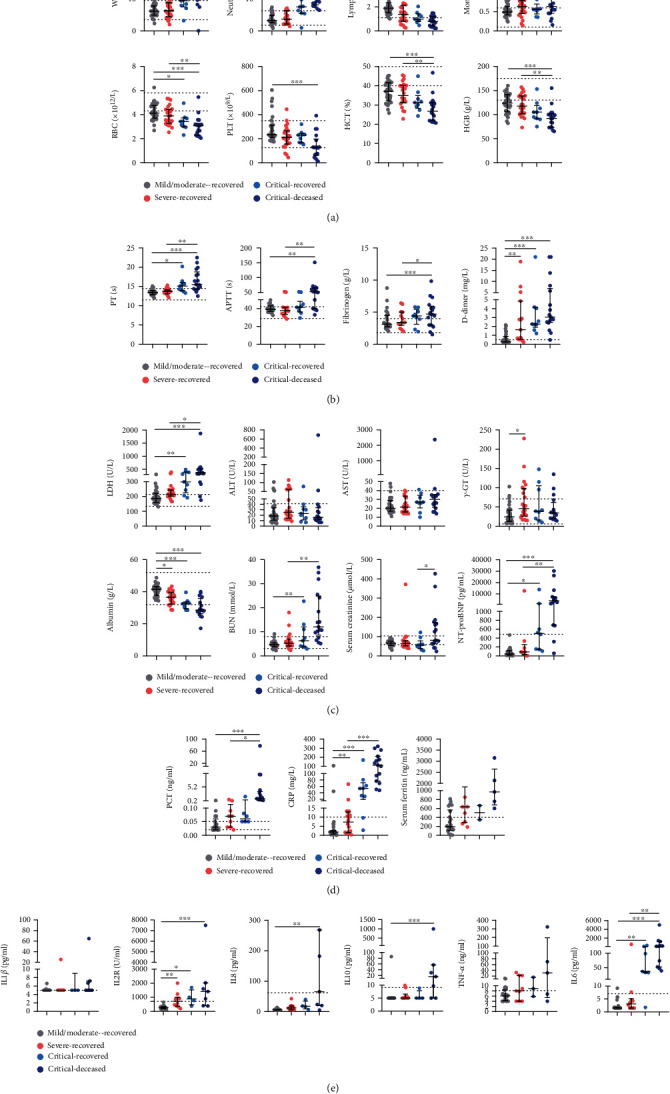
Comparison of clinical biomarkers between different groups of COVID-19 patients. 87 patients had relatively complete biochemical indicators as followings. Mild/moderate: *n* = 36, severe: *n* = 24, critical-recovered: *n* = 9, and critical-deceased patients: *n* = 18. The levels of these indicators were shown in medians and interquartile range. Differences between groups were analyzed using Kruskal-Wallis test and post hoc test (Dunn-Bonferroni). ^∗^*p* < 0.05; ^∗∗^*p* < 0.01; ^∗∗∗^*p* < 0.001. (a) Blood routine. (b) Coagulation function. (c) Liver, kidney and heart function. (d) Infection markers. (e) Cytokine profiles. PT: prothrombin time; APTT: activated partial thromboplastin time; LDH: lactate dehydrogenase; ALT: alanine aminotransferase; AST: aspartate aminotransferase; ALP: alkaline phosphatase; *γ*-GT: *γ*-glutamyl transpeptidase; NT-proBNP, amino-terminal probrain natriuretic peptide; CRP, C-reactive protein; IL-2R, IL-2 receptor.

To elucidate the role of nAb response during clinical progression, we first correlated a series of clinical biomarkers with the severity of illness to screen the indicators, which may be used as surrogates to reflect the magnitude of host inflammatory response and immunity. These biomarkers included lymphocytes, neutrophils, platelets (PLT), total protein, albumin, PT, APTT, D-dimer, LDH, AST, ALP, *γ*-GT, NT-proBNP, PCT, CRP, ferritin, IL-2R, IL-8, IL-10, TNF-*α*, and IL-6. Of four groups, critical-deceased patients had the highest levels of the measured biomarkers such as PT, APTT, D-dimer, LDH, AST, *γ*-GT, NT-proBNP, PCT, CRP, ferritin, IL-2R, IL-8, IL-10, TNF-*α*, and IL-6. In addition, the critical-deceased group also reported the lowest number of lymphocytes (Figure 4). Based on these data, our results suggest that the levels of the inflammatory biomarkers such as IL-6, CRP, and D-dimer might be related to the aggravation and deterioration of COVID-19, while lymphocytes might be used as a predictor of immune protection.

**Figure 5:**
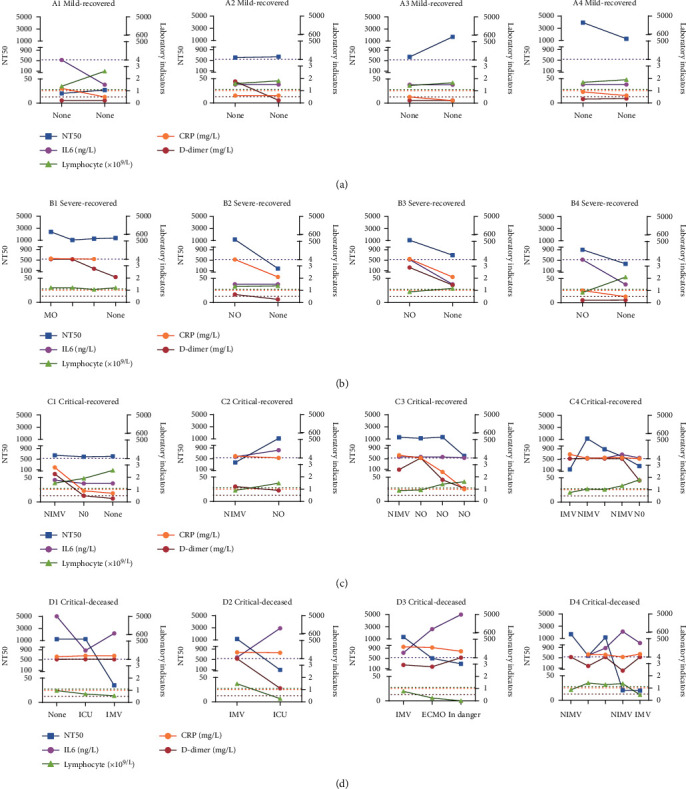
Longitudinal changes of neutralizing antibody response with clinical progression in representative patients. Four representative cases of mild/moderate, severe, critical-recovered, and critical-deceased groups were shown, respectively. nAb response was detected as previously described. The dynamic changes of nAb response, clinical biomarkers, and treatment regimens during clinical progression of different patients were, respectively, shown. NT50 with days after symptom onset was shown in the left axis, and laboratory indicators including IL6, CRP, D-dimer, and lymphocyte were shown in the right axis. The dotted lines with different colors represented the critical value for different biomarkers. IL6 (<7 ng/L), CRP (<1 mg/L), D-dimer (<0.5 mg/L), and lymphocyte (1.1 − 3.2 × 10^9^/L). (a) Mild/moderate patients. (b) Severe patients. (c) Critical-recovered patients. (d) Critical-deceased patients. None: no oxygen therapy; NO: nasal oxygen; MO: mask oxygen; NIMV: noninvasive mechanical ventilation; IMV: invasive mechanical ventilation; ECMO: extracorporeal membrane oxygenation.

Subsequently, four representative cases were, respectively, selected from mild/moderate, severe, critical-recovered, and critical-deceased groups (Figure 5, and more cases in Figure S1). Dynamic changes of nAb response for each patients during clinical progression were correlated with the above four biomarkers and treatment regimens. Mild/moderate patients did not need any kinds of oxygen therapy but recovered quickly with normal lymphocytes. There was no inflammatory reaction in this group. During hospitalization, the serum nAb response of 3 patients remained at a high level, and the NT50 value was higher than 1 : 500. Only A1 patient had a NT50 value of 1 : 20 (Figure 5(a)). Severe patients only had lymphocyte abnormalities in the acute attack stage and gradually recovered after inhaling nasal oxygen or mask oxygen. The nAb response of these patients declined gradually but maintained at a medium or high level with the recovery of the disease (Figure 5(b)). Critical patients suffered from severe dyspnea, low lymphocytes, and high levels of inflammatory biomarkers such as IL-6, CRP, and D-dimer. With the assistant treatment of noninvasive mechanical ventilation, these clinical biomarkers gradually restored to the normal level an d the nAb response remained at a medium or high level during hospitalization (Figure 5(c)). Critical-deceased patients could not prevent clinical progression to death, even with mechanical ventilation or ECMO support treatment. A continuous and intense storm of inflammatory cytokines was formed, resulting in the gradual decline of lymphocytes to very low levels (Figure 5(d)). As a result, the nAb response in their serum decreased sharply, eventually leading to the deterioration of the disease. Therefore, our results indicate that stable and high levels of serum nAb responses could prevent disease progression and deterioration.

## 4. Discussion

To explore the role of nAb response during clinical COVID-19 progression, dynamics of the nAb response in COVID-19 patients were correlated with disease severity and outcome in this study. We demonstrated that COVID-19 patients of different severity and outcome had differential dynamics of the nAb response over the course of disease. Age and disease severity of COVID-19 patients are the important factors that might affect significantly the magnitude of serum nAb response. Maintaining a high level of nAb response can prevent the deterioration of COVID-19, which highlights the protective role of nAb during clinical progression.

The early rising rate and the magnitude of nAb response in mild/moderate patients were quiet different to those of both severe and critical patients. Patients with various severities had differential viral loads, which might result in the difference of immune response [27, 39]. In line with other studies [40, 41], mild/moderate patients remained the normal immune response, which resulted in the relatively stable nAb response during hospitalization. On the contrary, strong inflammatory cytokine storm and inflammatory response resulted in the collapse of the immune system in critical-deceased patients, as demonstrated by the vanished lymphocytes and the sharp decline of nAb response. A reasonable explanation is that the generation and maintenance of nAb response in mild/moderate and severe patients might be associated with their normal immune system and function. In addition, our data also confirmed that other studies showed a decreased response of nAb in the patients over time. Several studies reported that nAb in convalescent patients persisted for 5 months [42] or longer [43, 44]. Although the levels of nAb following natural infection or vaccination influence the outcome of reexposure or infection, SARS-CoV-2-specific CD4^+^ and CD8^+^T cell responses are also relevant to the protection [45, 46]. Undoubtedly, fighting COVID-19 requires the coordinated participation of the entire adaptive immune systems.

Lymphopenia was associated with the disease deterioration [47]. In this study, severe and critical patients had more severe lymphopenia than mild/moderate patients, as also reported by other studies [48, 49]. SARS-CoV-2 showed the direct damage effect on lymphocytes [48, 49], which might result in lymphopenia. High levels of PT, APTT, FIB, and D-dimer were observed in severe and critical patients, which indicates the coagulation disorder. Because of high levels of LDH, *γ*-GT, albumin, urea nitrogen, creatinine, and NT-proBNP, critical patients had multiple organ injury. Biomarkers such as CRP and PCT [50, 51] suggested excessive inflammatory stress in vivo and the severe or critical illness of COVID-19 patients [52, 53]. Critical COVID-19 patients also showed high levels of several proinflammation cytokines, such as IL-2R, IL-8, and IL-6, and anti-inflammatory IL-10, as described by a previous report [54]. IL-6 is an important driver of the inflammatory cytokine storm, which might lead to acute lung injury, acute respiratory distress syndrome, and other tissue damages, progressing to multiple organ failure [55–57]. Consistent with other studies [40, 58], critical-deceased patients elevated the levels of IL-10, which might be responsible for the secondary infections, sepsis [55, 59], and T cell exhaustion [60–62]. Therefore, the severity of disease, more specifically, the impairment degree of lymphocyte function, attributes to the differential nAb responses.

Our findings have important implications for the control of COVID-19. Because of the protection effect of nAb response during disease progression, nAb should be used as an essential indicator for the prognosis of disease and the efficacy of vaccine. Although the nAb response in the serum of recipients immunized with the current emergency vaccines was detected, the titers of nAb varied with various factors such as the types of vaccines, detection methods of nAb, and geographical population. Our established neutralization assay based on the pseudotyped virus provides a good platform for comparing the levels of nAb response among different populations and different types of vaccines and warrants for the rapid screening of convalescent plasma donors and therapeutic monoclonal antibodies for the treatment of COVID-19.

## Data Availability

The data used to support the findings of this study are available from the corresponding authors upon request.

## Supplementary Materials

Supplementary Materials Table S1: characteristics of 123 COVID-19 patients. Table S2: characteristics of 68 COVID-19 patients. Table S3: characteristics of 42 COVID-19 patients with serial serum samples. Figure S1: longitudinal changes of neutralizing antibody response with clinical progression in more representative patients.
