# Supplementary material for: Kinetics of Neutralizing Antibody Response Underscores Clinical COVID-19 Progression
Source: J Immunol Res. 2021 Oct 19;2021:9822706. doi: 10.1155/2021/9822706 (PMC8548120; doi:10.1155/2021/9822706)
Supplement: Supplementary Materials — Table S1: characteristics of 123 COVID-19 patients. Table S2: characteristics of 68 COVID-19 patients. Table S3: characteristics of 42 COVID-19 patients with serial serum samples. Figure S1: longitudinal changes of neutralizing antibody response with clinical progression in more representative patients. [file 9822706.f1.docx]

**Supplements**

| **Table S1. Characteristics of 123 COVID-19 patients** | | |
| --- | --- | --- |
| COVID-19 Patients | | N=123 |
| Sex | Female | 60 |
|  | Male | 63 |
| Age | <60 | 61 |
|  | >=60 | 62 |
| Severity/Outcome | Mild/moderate-recovered | 68 |
|  | Severe-recovered | 26 |
|  | Critical-recovered | 11 |
|  | Critical-deceased | 18 |

| **Table S2. Characteristics of 68 COVID-19 patients** | | |
| --- | --- | --- |
| COVID-19 Patients | | N=68 |
| Sex | Female | 33 |
|  | Male | 35 |
| Age | <60 | 28 |
|  | >=60 | 40 |
| Severity | Mild/moderate-recovered | 27 |
|  | Severe-recovered | 17 |
|  | Critical-recovered | 9 |
|  | Critical-deceased | 15 |

| **Table S3. Characteristics of 42 COVID-19 patients** | | |
| --- | --- | --- |
| COVID-19 Patients | | N=42 |
| Sex | Female | 19 |
|  | Male | 23 |
| Age | <60 | 12 |
|  | >=60 | 30 |
| Severity | Mild/moderate-recovered | 8 |
|  | Severe-recovered | 11 |
|  | Critical-recovered | 8 |
|  | Critical-deceased | 15 |


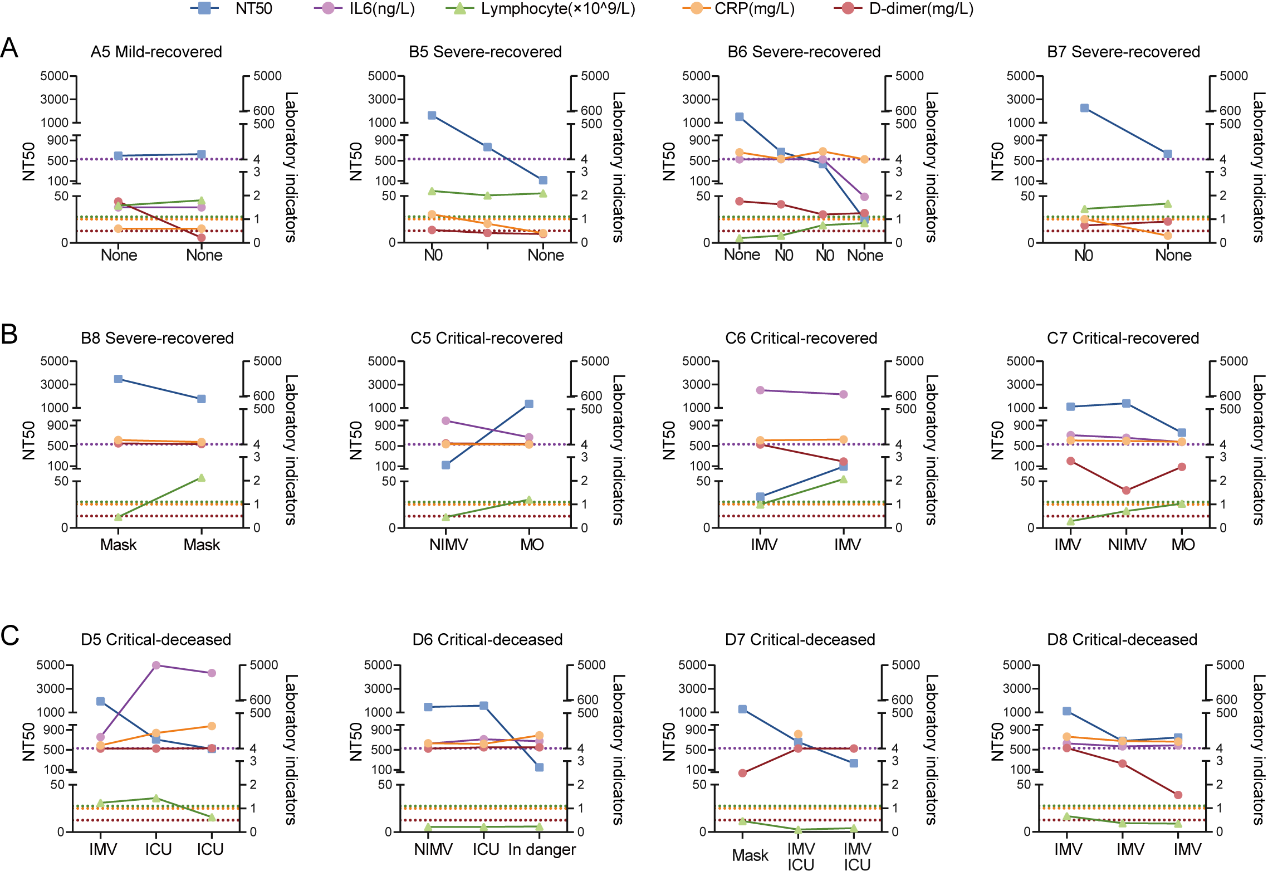
 **Figure S1. Longitudinal changes of neutralizing antibody response with clinical progression in more representative patients.**

NT50 with days after symptom onset was shown in the left axis and laboratory indicators including IL6, CRP D-dimer, and Lymphocyte were shown in the right axis. The dotted lines with different colors represented the critical value for different biomarkers. IL6 (<7ng/L), CRP (<1 mg/L), D-dimer (<0.5mg/L), and Lymphocye (1.1-3.2 x10^9/L). None, no oxygen therapy; NO, nascal oxygen; MO, mask oxygen; NIMV, Non-invasive mechanical ventilation; IMV, Invasive mechanical ventilation; ECMO, extracorporeal membrane oxygenation.
